# CRM197 reverses paclitaxel resistance by inhibiting the NAC‐1/Gadd45 pathway in paclitaxel‐resistant ovarian cancer cells

**DOI:** 10.1002/cam4.2512

**Published:** 2019-09-06

**Authors:** Xiao‐han Tang, Hui Li, Xiu‐shuang Zheng, Mei‐song Lu, Yuan An, Xiao‐lei Zhang

**Affiliations:** ^1^ Department of Gynecology and Obstetrics The First Affiliated Hospital of Harbin Medical University Harbin China

**Keywords:** CRM197, HB‐EGF, NAC‐1, ovarian cancer, paclitaxel resistance

## Abstract

Heparin‐binding epidermal growth factor‐like growth factor (HB‐EGF) is a new promising target for the treatment of ovarian cancer. Our previous study showed that cross‐reacting material 197 (CRM197), a specific HB‐EGF inhibitor, significantly reverses resistance against paclitaxel in paclitaxel‐resistant ovarian cancer cells. However, the mechanism of the effect of CRM197 on the reversion of paclitaxel resistance was unclear. In this study, in vitro and in vivo data suggested that CRM197 treatment sensitized paclitaxel‐resistant ovarian cancer cells to paclitaxel, at least in part, via nucleus accumbens‐1 (NAC‐1) and its downstream pathway, DNA damage‐inducible 45‐γ interacting protein (Gadd45gip1)/growth arrest and DNA damage‐inducible 45 (Gadd45), in A2780/Taxol and SKOV3/Taxol cells. The results also showed that CRM197 activated the proapoptotic JNK/p38MAPK pathway to enhance caspase‐3 activity and apoptosis by downregulation of the NAC‐1/Gadd45gip1/Gadd45 pathway, leading to reversion of paclitaxel resistance in A2780/Taxol and SKOV3/Taxol cells. This study provides the first mechanism through which CRM197 significantly reverses resistance against paclitaxel by modulating the NAC‐1/Gadd45gip1/Gadd45 pathway in paclitaxel‐resistant ovarian cancer cells, and the mechanism of HB‐EGF inhibition as a novel therapeutic strategy for patients with paclitaxel‐resistant ovarian cancer.

## Graphical abstract

CRM197 treatment sensitized the paclitaxel‐resistant ovarian cancer to paclitaxel, at least in part, via NAC‐1 and its downstream Gadd45gip1/ Gadd45 pathway in A2780/Taxol and SKOV3/Taxol cells. The data also shown that CRM197 activates the proapoptotic JNK/p38MAPK pathway to enhance caspase‐3 activity and cell apoptosis by the downregulation of NAC‐1/Gadd45gip1/Gadd45 pathway, resulting in the reversion of paclitaxel resistance in A2780/Taxol and SKOV3/Taxol cells. This study provides the first mechanism through which CRM197 significantly reverses resistance against paclitaxel by modulating the NAC‐1/Gadd45gip1/Gadd45 pathway in paclitaxel‐resistant ovarian cancer cells, and the mechanism of HB‐EGF inhibition as a novel therapeutic strategy for patients with paclitaxel‐resistant ovarian cancer.

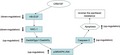

## 1 INTRODUCTION

Currently, ovarian cancer is the most lethal gynecological malignancy. Paclitaxel has been widely used as a frontline therapeutic agent for ovarian cancer.^1^ However, nearly all patients with ovarian cancer, who initially respond to paclitaxel, relapse and become refractory to chemotherapy. Moreover, therapies developed over the last 30 years have not improved survival rates. Patients who relapse or do not initially respond to traditional chemotherapy are thought to have drug‐resistant cancer cells, resulting in cancer relapse and lethality.^2^ To develop a novel effective therapy to restore the chemosensitivity of patients with ovarian cancer, further understanding of the molecules and mechanisms leading to paclitaxel resistance of ovarian cancer is needed.

Heparin‐binding epidermal growth factor‐like growth factor (HB‐EGF) is one of the seven ligands of the epidermal growth factor receptor (EGFR) and the primary EGFR ligand altered in ovarian cancer.^3^, ^4^ HB‐EGF plays a critical role in proliferation, angiogenesis, and metastasis of ovarian cancer, making it a putative therapeutic target for ovarian cancer. Cross‐reacting material 197 (CRM197), an HB‐EGF inhibitor, is a nontoxic mutant of diphtheria toxin, which shares immunological properties with the native molecule.^5^, ^6^ CRM197 binds to the soluble form of HB‐EGF as well as pro‐HB‐EGF and blocks mitogenic activity by inhibiting the binding between EGFR and HB‐EGF.^7^ Although CRM197 does not inhibit the mitogenic activity of other EGFR ligands, it is a specific inhibitor of HB‐EGF and the only known inhibitor that can be used for cancer therapy in mice and humans.^8^

Our previous in vitro and in vivo study demonstrated that CRM197 significantly reverses resistance against paclitaxel in paclitaxel‐resistant ovarian cancer cells.^9^ However, the mechanism of the reversion of paclitaxel resistance by CRM197 was unclear. In this study, we investigated the mechanism of CRM197 in alleviating paclitaxel resistance. First, we investigated the role of nucleus accumbens‐1 (NAC‐1) and its downstream pathway, growth arrest and DNA damage‐inducible 45‐γ interacting protein (Gadd45gip1)/growth arrest and DNA damage‐inducible 45 (Gadd45), in CRM197‐mediated reversal of paclitaxel resistance in ovarian cancer cells. Second, we further investigated regulation of the MAPK pathway by the NAC‐1/Gadd45gip1/Gadd45 pathway following treatment of ovarian cancer cells with CRM197. Third, we examined the effect of activation of the MAPK pathway by CRM197 on the caspase‐3 activity and cell viability.

## 2 METHODS

### 2.1 Cell culture and CRM197 treatment

Human parental ovarian cancer cell lines (A2780 and SKOV3) and paclitaxel‐resistant cell lines (A2780/Taxol and SKOV3/Taxol) were generously provided by Dr Lan Xiao (Department of Gynecology and Obstetrics, Third Affiliated Hospital of Sun Yan‐sen University, Guangzhou, China) and cultured as described previously.^10^ CRM197 (Sigma‐Aldrich) treatment of parental and paclitaxel‐resistant ovarian cancer cells was performed as described previously.^10^

### 2.2 Real‐time PCR

Real‐time PCR was performed using SYBR® Premix Ex Taq™ II (TaKaRa Biotechnology) in an Applied Biosystems 7500 Real‐Time PCR System as described previously.^11^ The human *beta‐actin* gene was used as an internal control. Primers for *NAC‐1* (accession no. NM_052876.3) and *beta‐actin* (GenBank accession no. NM_001101.3) were as follows. *NAC‐1*, sense 5′‐AAGCTGAGGATCTGCTGGAA‐3′, antisense 5′‐CCAGACACTGCAGATGGAGA‐3′; *beta‐actin*, sense 5′‐CCGTAAAGACCTCTATGCCAACA‐3′, antisense 5′‐CGGACTCATCGTACTCCTGCT‐3′. PCR was conducted under the following conditions: 30 seconds at 95°C followed by 40 cycles of 5 seconds at 95°C and 35 seconds at 60°C. The $\Delta^{\Delta C_{t}}$method was used for normalization and to determine fold changes.

### 2.3 MTT assay

The IC_50_ of paclitaxel (Sigma‐Aldrich, St Louis, MO) was determined by a 3‐(4,5‐dimethylthiazol‐2‐yl)‐2,5‐diphenyl‐tetrazolium bromide (MTT) assay as described previously.^11^ Cells were incubated for 48 hours with various concentrations of paclitaxel (0, 0.125, 0.25, 0.5, 1, 2, 4, and 8 μmol/L). Absorbance was determined using a 96‐well Microplate Reader (ELX800; Bio‐Tek) at 490 nm.

### 2.4 Caspase‐3 activity assay

The caspase‐3 activity assay was performed using a colorimetric activity assay kit (R&D Systems), according to the manufacturer's instructions. Cells cultured with or without CRM197 were lysed, and total protein was measured by the Bradford assay. Samples were then analyzed for caspase‐3 activity by Ac‐DEVD as described previously.^11^ Absorbance was measured at 405 nm in the ELX800 plate reader.

### 2.5 Western blot analysis

Whole cell lysates (50 mg protein) were used for western blotting as described previously.^12^ The proteins were separated by SDS‐PAGE and then transferred to nitrocellulose membranes. The blot was probed with an anti‐NAC‐1 monoclonal antibody (Abcam PLC) at a 1:100 dilution, anti‐Gadd45gip1 polyclonal antibody (Abcam PLC) at a 1:100 dilution, anti‐p38 MAPK or ‐JNK monoclonal antibodies (Santa Cruz), and anti‐phospho‐p38 MAPK or anti‐phospho JNK polyclonal antibodies (Abcam PLC). Antibody binding was detected using an enhanced chemiluminescence detection reagent (Amersham Biosciences), according to the manufacturer's protocol.

### 2.6 NAC‐1 short hairpin RNA (shRNA) and Gadd45gip1 small interfering RNA (siRNA) transfections

shRNA against NAC‐1 and siRNA against Gadd45gip1 were purchased from Dharmacon Inc. Cells were grown to 80% confluence, and then NAC‐1 shRNA, Gadd45gip1 siRNA, or scramble control siRNA were transfected into cells with or without CRM197 treatment using OligofectAMINE 2000 (Invitrogen) as described previously.^11^ Western blotting was performed to examine the silenced protein levels after incubation for 48 hours.

### 2.7 Immunohistochemistry

Immunohistochemical staining of CRM197‐treated and ‐untreated A2780, A2780/Taxol, and NAC‐1 shRNA A2780/Taxol cells was performed using the anti‐NAC‐1 monoclonal antibody. Staining intensity was scored using a 4‐tiered scale, 0 (undetectable), 1+ (weakly positive), 2+ (moderately positive), and 3+ (intensely positive), based on the staining intensity and proportion of stained cells as described previously.^13^

### 2.8 Xenografts

Mice were randomly divided into A2780, A2780/Taxol, and NAC‐1 shRNA A2780/Taxol groups (eight mice/group). PBS (200 μL) containing 1 × 10^7^ A2780, A2780/Taxol, or NAC‐1 shRNA1 A2780/Taxol cells was subcutaneously injected into female BALB/c nude mice (Vital River Laboratory). The tumor volume was calculated by the formula 0.5 × width^2^ × length. After the mean tumor size reached 100 mm^3^, A2780 and A2780/Taxol groups were randomly subdivided into CRM197 and control groups (four mice/group). After 4 weeks of CRM197 treatment, CRM197 dissolved in 200 μL PBS (1 mg/wk) was injected intraperitoneally into CRM197 groups (five mice/group) each week. The control groups (four mice/group) were injected intraperitoneally with 200 μL PBS for 4 weeks. Then, the mice were sacrificed and their tumors were subjected to immunohistochemical staining. All animal procedures were approved by the Committee on the Ethics of Harbin Medical University and complied with the Guidelines for the Care and Use of Laboratory Animals of Harbin Medical University.

### 2.9 In vivo imaging

To visualize intraperitoneal tumors in mice, 100 mg/mL CRM197 was injected intraperitoneally, and macroscopic in vivo fluorescence and luminescence imaging was carried out using an IVIS system. A fresh solution of D‐luciferin (OZ Biosciences) was prepared at 15 mg/mL in DPBS. For in vivo imaging, 48 hours after administration of the photosensitizer, the mice were injected intraperitoneally with 150 mg D‐luciferin/kg body weight at 10‐15 minutes before imaging.^14^, ^15^ Optical images obtained by the IVIS were analyzed with Living Image Software.

### 2.10 Statistical analysis

Data are presented as means ± SD of three independent experiments. The Student's t‐test was used for statistical analyses (Sigmastat v. 3.5 software). A *P*‐value of <.05 was considered as significant.

## 3 RESULTS

### 3.1 CRM197 enhances caspase‐3 activity via the JNK/p38MAPK pathway

**Figure 1:**
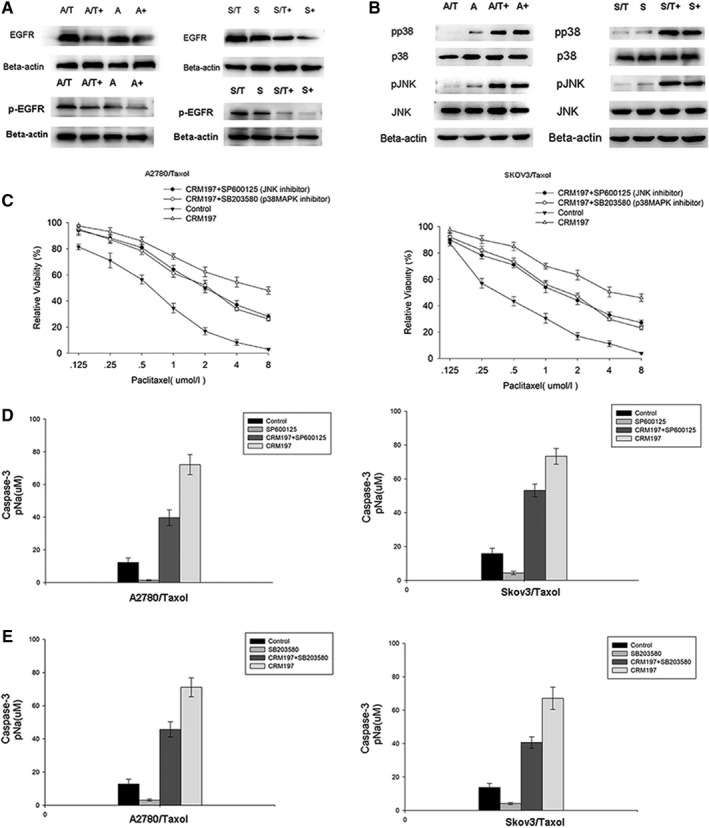
CRM197 enhances caspase‐3 activity via the JNK/p38MAPK pathway (A) CRM197 decreased EGFR expression in parental (A2780 and SKOV3) and paclitaxel‐resistant (A2780/Taxol and SKOV3/Taxol) ovarian cancer cells. A/T, A2780/Taxol; A, A2780; A/T+, A2780/Taxol+CRM197; A+, A2780+CRM197; S/T, SKOV3/Taxol; S/T+, SKOV3/Taxol+CRM197; S, SKOV3; S+, SKOV3+CRM197. (B) CRM197 induced alterations in JNK/p38MAPK signaling of ovarian cancer cells. Activation statuses of JNK and p38 MAPK were examined by western blot analysis in CRM197‐treated and ‐untreated parental (A2780 and SKOV3) and paclitaxel‐resistant (A2780/Taxol and SKOV3/Taxol) ovarian cancer cells. (C) Sensitivities to paclitaxel in A2780/Taxol and SKOV3/Taxol cells treated with CRM197 (100 μg/mL), CRM197+JNK inhibitor (SP600125), CRM197+p38MAPK inhibitor (SB203580), or saline (control) were determined by MTT assays. Cells were treated with paclitaxel at various concentrations (0, 0.125, 0.25, 0.5, 1, 2, 4, and 8 μmol/L) for 48 h. (D) Caspase‐3 activity in A2780/Taxol and SKOV3/Taxol cells treated with saline (control), CRM197, SP600125 (JNK inhibitor), or CRM197+SP600125. (E) Caspase‐3 activity in A2780/Taxol and SKOV3/Taxol cells treated with saline, CRM197, SB203580 (p38MAPK inhibitor), or CRM197+SB203580

EGFR is the critical signal transducer linking HB‐EGF to MAPK cascades.^16^ Our previous study showed that HB‐EGF inhibitor CRM197 significantly suppresses the expression of EGFR in ovarian cancer cells.^9^ To determine whether the EGFR/JNK/p38MAPK pathway was affected by CRM197 treatment in ovarian cancer cells, we examined EGFR expression and JNK/p38MAPK pathway activity in ovarian cancer cells following CRM197 treatment. CRM197 significantly induced downregulation of EGFR expression (Figure 1A) and activation of JNK/p38MAPK (Figure 1B) in parental (A2780 and SKOV3) and paclitaxel‐resistant (A2780/Taxol and SKOV3/Taxol) ovarian cancer cells (both *P* < .01), suggesting that CRM197 treatment activates the JNK/p38MAPK pathway by inhibiting EGFR expression. The increased expression pf EGFR and activation of JNK and p38MAPK in A2780/Taxol and SKOV3/Taxol cells were much higher than those in A2780 and SKOV3 cells, suggesting that activation of JNK and p38 MAPK is related to paclitaxel resistance.

To further examine whether activation of JNK/p38MAPK is involved in CRM197‐mediated reversal of paclitaxel resistance, we evaluated cell viability (Figure 1C) and the 50% inhibitory concentrations (IC_50_) of paclitaxel (Table 1) in A2780/Taxol and SKOV3/Taxol cells treated with saline (control group), CRM197, CRM197+SP600125 (JNK inhibitor), or CRM197+SB203580 (p38MAPK inhibitor) using MTT assays. The results indicated 6.22‐, 2.37‐, and 2.52‐fold reductions in paclitaxel resistance of A2780/Taxol cells and 5.98‐, 2.21‐, and 2.48‐fold reductions in paclitaxel resistance of A2780/Taxol cells treated with CRM197, CRM197+SP600125, and CRM197+SB203580 compared with control A2780 and SKOV3 cells, respectively (Table 1, all *P* < .01). The viability of A2780/Taxol and SKOV3/Taxol cells treated with CRM197+SP600125 and CRM197+SB203580 was significantly higher than that of A2780/Taxol and SKOV3/Taxol cells treated with CRM197 (Figure 1C, both *P* < .01). These data suggest that CRM197 effectively restores the sensitivity of paclitaxel‐resistant cells to paclitaxel and induces apoptosis by activation of JNK/p38 MAPK signaling.

**Table 1:** The role of JNK/p38MAPK played in the CRM197‐mediated reversal of the paclitaxel‐resistant phenotype in paclitaxel‐resistant ovarian cancer cells

Groups	Paclitaxel IC_50_ (μmol/L)			
	A2780	A2780/Taxol	SKOV3	SKOV3/Taxol
Saline (control)	0.811 ± 0.104	38.261 ± 0.624	0.835 ± 0.112	37.672 ± 0.575
CRM197 (100 μg/mL)	0.621 ± 0.085	6.147 ± 0.135* (6.22)	0.677 ± 0.096	6.300 ± 0.135* (5.98)
CRM197 (100 μg/mL)+SP600125	0.771 ± 0.093	16.143 ± 0.368*, # (2.37)	0.812 ± 0.098	17.046 ± 0.368*, # (2.21)
CRM197 (100 μg/mL)+SB203580	0.756 ± 0.089	15.182 ± 0.329*, # (2.52)	0.792 ± 0.084	15.190 ± 0.329*, # (2.48)

The fold reversal of drug resistance (number in parentheses) = IC_50(treatments)_/IC_50(control)_. Data are presented as mean ± SD from three independent experiments.

* *P* < .01 vs the control paclitaxel‐resistant cells.

^#^ *P* < .01 vs the paclitaxel‐resistant cells with CRM197 treatment.

To confirm that the JNK/p38 MAPK pathway initiates a proapoptotic pathway in response to CRM197, we examined caspase‐3 activity in A2780/Taxol and SKOV3/Taxol cells treated with saline, CRM197, SP600125, CRM197+SP600125, or SB203580, CRM197+SB203580 (Figure 1D,E). A2780/Taxol cells treated with CRM197+SP600125 (Figure 1D) and CRM197+SB203580 (Figure 1E) exhibited dramatic increases in caspase‐3 activity by 31.2% and 25.5%, respectively, compared with A2780/Taxol cells treated with CRM197 (both *P* < .01). SKOV3/Taxol cells treated with CRM197+SP600125 (Figure 1D) and CRM197+SB203580 (Figure 1E) showed dramatic decreases in caspase‐3 activity of 30.4% and 24.7%, respectively, compared with SKOV3/Taxol cells treated with CRM197 (both *P* < .01). These results demonstrated that the JNK/p38MAPK pathway induced a proapoptotic pathway that contributed to CRM197‐mediated reversal of paclitaxel resistance in A2780/Taxol cells.

### 3.2 Effect of CRM197 treatment on NAC‐1 expression

NAC‐1, a tumor recurrence‐associated gene, has important roles in cell survival and apoptosis by modulating apoptosis‐related pathways.^17^, ^18^ However, the role of NAC‐1 in CRM197‐mediated reversal of paclitaxel resistance remains largely unclear. To elucidate the mechanism of CRM197 in reversion of paclitaxel resistance by regulation of the proapoptotic pathway, we first investigated the relationship between NAC‐1 and CRM197.

**Figure 2:**
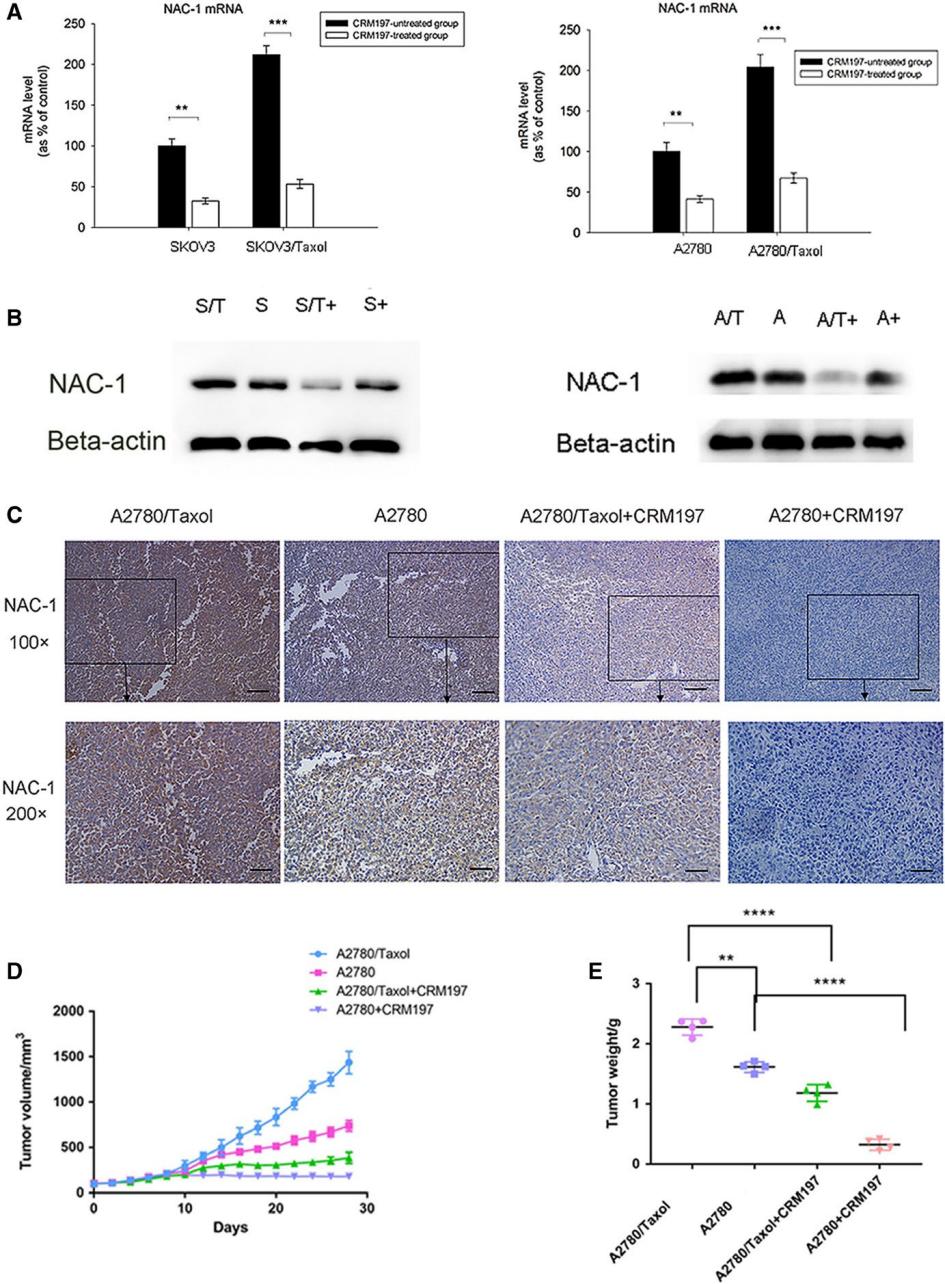
CRM197 inhibits paclitaxel resistance by downregulation of NAC‐1 expression in ovarian cancer cells and xenografts. A and B, CRM197 decreased NAC‐1 expression in parental (A2780 and SKOV3) and paclitaxel‐resistant (A2780/Taxol and SKOV3/Taxol) ovarian cancer cells. Expression of NAC‐1 mRNA (A) and protein (B) in CRM197‐treated and ‐untreated cells was measured by real‐time PCR and western blot analysis, respectively. **P < .01; ***P < .001 vs CRM197‐untreated cells. β‐Actin was used as an endogenous control. C, Immunoreactivity of NAC‐1 following CRM197 treatment in xenografted A2780 and A2780/Taxol tumors was examined by immunohistochemistry. Scale bar, 100 μm. D and E, Tumor size (D) and tumor weight (E) of xenografted A2780 and A2780/Taxol tumors following CRM197 treatment was monitored and measured each week. Tumor volume was calculated using the equation *x*2*y*/2 (where *x* < *y*). Tumor weight was measured at completion of the experiment

We measured NAC‐1 expression after CRM197 treatment of parental (A2780 and SKOV3) and paclitaxel‐resistant (A2780/Taxol and SKOV3/Taxol) ovarian cancer cells. The expression levels of NAC‐1 mRNA (Figure 2A) and protein (Figure 2B) in A2780/Taxol and SKOV3/Taxol cells were significantly higher than those in A2780 and SKOV3 cells (*P* < .01), suggesting that NAC‐1 contributes to paclitaxel resistance of ovarian cancer cells. We found that CRM197 significantly downregulated the expression of NAC‐1 mRNA (Figure 2A) and protein (Figure 2B) in parental (A2780 and SKOV3) and paclitaxel‐resistant (A2780/Taxol and SKOV3/Taxol) cells (A2780 and SKOV3 cells, *P* < .01; A2780/Taxol and SKOV3/Taxol cells, *P* < .001).

To confirm the results, we established A2780 and A2780/Taxol xenografted mice. Two weeks after subcutaneous injection, enhanced tumorigenicity was observed in paclitaxel‐resistant ovarian tumors. During 4 weeks of CRM197 treatment, the tumor mass was measured every week, and then the mice were sacrificed and their tumors were collected for weight measurement. We examined NAC‐1 protein expression by immunohistochemistry (Figure 2C), and the tumor volume (Figure 2D) and tumor weight (Figure 2E) in A2780 and A2780/Taxol xenografts following CRM197 treatment. The staining intensity of NAC‐1 in CRM197‐untreated A2780/Taxol tumors was 3+, whereas the staining intensity of NAC‐1 in CRM197‐treated A2780/Taxol tumors was 1+. The staining intensity of NAC‐1 in CRM197‐untreated A2780 tumors was 2+, whereas the staining intensity of NAC‐1 in CRM197‐treated A2780 tumors was close to 0. Similar to the in vitro data, CRM197 effectively suppressed NAC‐1 expression and the formation of A2780/Taxol tumors compared with A2780 tumors in vivo. A2780/Taxol cells, which expressed higher levels of NAC‐1, formed larger tumors than A2780 cells.

### 3.3 NAC‐1 silencing enhances the effect of CRM197 on tumor growth

**Figure 3:**
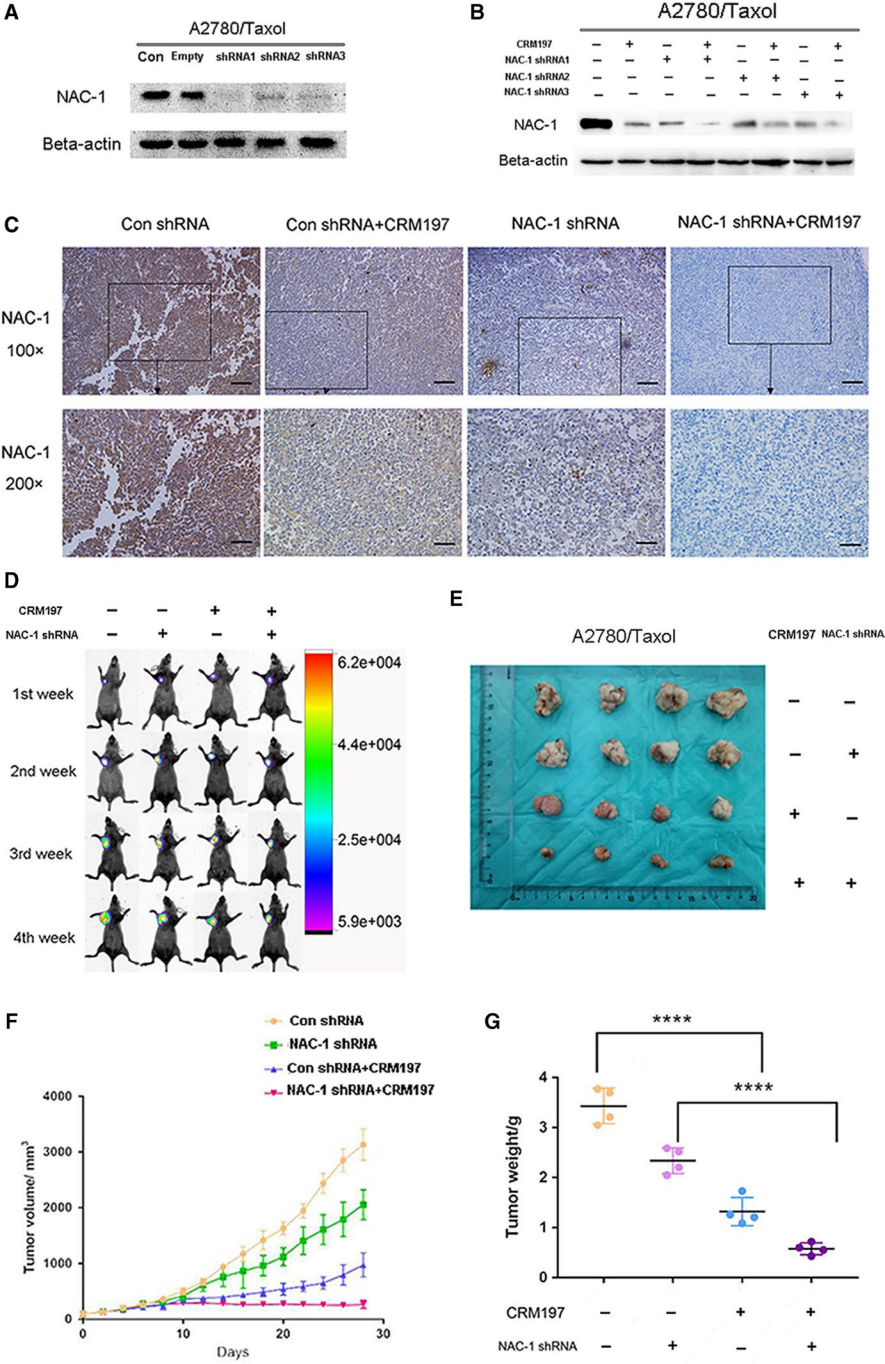
Inhibition of NAC‐1 expression enhances the effect of CRM197 on resistance against paclitaxel. A, A2780/Taxol cells were transiently transfected with NAC‐1 shRNA (NAC‐1 shRNA1, shRNA2, and shRNA3) or scramble shRNA (Empty), or treated with PBS (Con, control). Western blotting for NAC‐1 was performed to detect the inhibition efficiency of shRNA. B, NAC‐1 expression was examined by western blotting in A2780/Taxol cells treated with NAC‐1 shRNA (NAC‐1 shRNA1, shRNA2, and shRNA3) or control shRNA following CRM197 treatment. C, Immunoreactivity of NAC‐1 following CRM197 treatment in xenografts of A2780/Taxol cells transfected with control shRNA or NAC‐1 shRNA (NAC‐1 shRNA1) was examined by immunohistochemistry. Scale bar, 100 μm. D, Fluorescence imaging of mice treated with NAC‐1 shRNA (NAC‐1 shRNA1) or control shRNA following CRM197 treatment. E‐G, Tumor size (E, F) and tumor weight (G) of xenografts of A2780/Taxol cells transfected with control and NAC‐1 shRNA (NAC‐1 shRNA1) following CRM197 treatment was monitored and measured each week. Tumor volume was calculated using the equation *x*2*y*/2 (where *x* < *y*)

To confirm the role of NAC‐1 in the effects of CRM197, we transiently transfected A2780/Taxol cells with shRNA against NAC‐1 (NAC‐1 shRNA1, shRNA2, and shRNA3) or control shRNA and examined their silencing effect by western blotting (Figure 3A). Then, the NAC‐1 expression level in response to CRM197 treatment was analyzed by western blotting in A2780/Taxol cells transfected with NAC‐1 shRNA or control shRNA. Both CRM197 treatment and NAC‐1 shRNA transfection (NAC‐1 shRNA1, shRNA2, and shRNA3) significantly inhibited NAC‐1 expression compared with the control shRNA (Figure 3B, all *P* < .01). These data suggested that NAC‐1 contributed to CRM197‐mediated reversal of paclitaxel resistance. We next established A2780/Taxol NAC‐1 shRNA (NAC‐1 shRNA1) xenografted mice. First, we examined NAC‐1 protein expression in xenografts following CRM197 treatment by immunohistochemical staining (Figure 3C). Then, we detected the luminescent signals by fluorescence imaging (Figure 3D) and measured the tumor size (Figure 3E,F) and tumor weight (Figure 3G) of control shRNA and NAC‐1 shRNA A2780/Taxol xenografts following CRM197 treatment. The staining intensity of NAC‐1 in CRM197‐untreated control shRNA A2780/Taxol tumors was 3+, whereas the staining intensity of NAC‐1 in CRM197‐treated control shRNA A2780/Taxol tumors was 1+. The staining intensity of NAC‐1 in CRM197‐untreated NAC‐1 shRNA A2780/Taxol tumors was 2+, whereas the immunointensity of NAC‐1 in CRM197‐treated NAC‐1 shRNA A2780/Taxol tumors was close to 0. Similar to the in vitro data, the in vivo results showed that inhibition of NAC‐1 expression by shRNA enhances the reversion of paclitaxel resistance by CRM197, suggesting that CRM197 significantly reverses resistance against paclitaxel by inhibiting NAC‐1 expression in ovarian cancer cells.

### 3.4 NAC‐1 overexpression or silencing affects CRM197‐mediated cell proliferation and caspase‐3 activity

**Figure 4:**
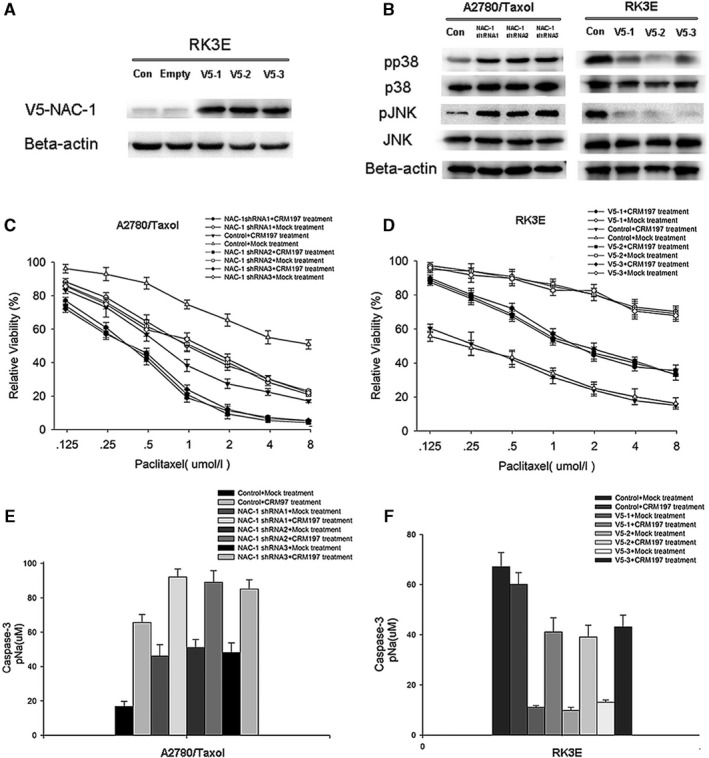
CRM197 activates the proapoptotic JNK/p38MAPK pathway by downregulation of NAC‐1 expression in A2780/Taxol cells. A, RK3E cells were transfected with NAC‐1 tagged with V5 (V5‐1, V5‐2, and V5‐3) or scramble siRNA (Empty) or treated with PBS (Con, control). Western blotting for V5‐NAC‐1 was performed to detect the knockdown efficiency. B, Activation statuses of JNK and p38 MAPK were examined by western blotting in A2780/Taxol cells treated with NAC‐1 shRNA (NAC‐1 shRNA1, shRNA2, and shRNA3) or control empty shRNA and in normal epithelial RK3E cells with undetectable NAC‐1 expression treated with NAC‐1 tagged with V5 (V5‐1, V5‐2, and V5‐3) or the empty vector control. C and E, A2780/Taxol cells transfected with NAC‐1 shRNA (NAC‐1 shRNA1, shRNA2, and shRNA3) or control shRNA were treated with CRM197 or dimethyl sulfoxide (Mock treatment), and cell viability rates (C) and caspase‐3 activity (E) in response to paclitaxel treatment were determined (*P* < .05). D and F, RK3E cells transfected with the V5‐NAC‐1 vector (V5‐1, V5‐2, and V5‐3) or control vector were treated with CRM197 or dimethyl sulfoxide (Mock treatment), and cell viability rates (D) and caspase‐3 activity (F) in response to paclitaxel were determined (*P* < .05)

The above findings suggest that CRM197 not only mediates the proapoptotic JNK/p38MAPK pathway, but also downregulates NAC‐1 expression. To investigate whether CRM197 activates the proapoptotic JNK/p38MAPK pathway by regulating NAC‐1, we used two independent but complementary approaches. First, we ectopically expressed NAC‐1 tagged with V5 (V5‐1, V5‐2, and V5‐3) in normal epithelial cell line RK3E with undetectable NAC‐1 expression and analyzed the change in activation of the JNK/p38MAPK pathway (Figure 4A,B, both *P* < .05). RK3E cells are insensitive to taxol, and we found low expression of NAC‐1 in RK3E cells (Figure 4A). Activation of the JNK/p38MAPK pathway was significantly reduced in RK3E cells transfected with the V5‐NAC‐1 vector compared with control vector‐transfected cells (Figure 4B). Second, we observed a decrease in the NAC‐1 expression level with a concomitant increase in activation of the JNK/p38MAPK pathway in A2780/Taxol cells (Figure 4B, *P* < .05). These data revealed that CRM197 activates the JNK/p38MAPK pathway by downregulation of NAC‐1 expression.

To determine whether NAC‐1 expression contributes to CRM197‐mediated reversal of paclitaxel resistance by activating the proapoptotic JNK/p38MAPK pathway, we examined cell viability and caspase‐3 activity in A2780/Taxol cells transfected with NAC‐1 shRNA (NAC‐1 shRNA1, shRNA2m and shRNA3) and RK3E cells transfected with V5‐NAC‐1 (V5‐1, V5‐2, and V5‐3). Inhibition of NAC‐1 by NAC‐1 shRNA significantly suppressed cell viability rates (Figure 4C) and enhanced caspase‐3 activity (Figure 4E) in A2780/Taxol cells with NAC‐1 shRNA+Mock treatment compared with those in A2780/Taxol cells with control shRNA+Mock treatment (both *P* < .05), whereas CRM197 significantly suppressed cell viability rates (Figure 4C) and enhanced caspase‐3 activity (Figure 4E) in A2780/Taxol cells with NAC‐1 shRNA+CRM197 treatment compared with A2780/Taxol cells with NAC‐1 shRNA+Mock treatment (*P* < .05). As shown in Figure 4D,F, the cell viability rate (Figure 4D) of V5‐NAC‐1 RK3E cells with Mock treatment was much higher than that of control RK3E cells with Mock treatment (*P* < .05). In addition, caspase‐3 activity (Figure 4F) in V5‐NAC‐1 RK3E cells with Mock treatment was much lower than that in control RK3E cells with Mock treatment (*P* < .05). CRM197 treatment of V5‐NAC‐1 RK3E cells significantly increased the cell viability rate (Figure 4D) and inhibited caspase‐3 activity (Figure 4F) compared with control RK3E cells with Mock treatment (*P* < .05). As shown in Table 2, CRM197 treatment induced 4.76‐, 4.60‐, and 4.64‐fold reductions in paclitaxel resistance of RK3E cells transfected with V5‐NAC‐1 (V5‐1, V5‐2, and V5‐3 respectively) and treated with CRM197 compared with cells without CRM197 treatment. These results confirmed that CRM197‐mediated reversal of paclitaxel resistance activates the JNK/p38MAPK pathway by inhibition of NAC‐1 expression.

**Table 2:** The role of NAC‐1 played in the CRM197‐mediated reversal of the paclitaxel‐resistant phenotype in RK3E cells

Groups	Paclitaxel IC_50_ (μmol/L)	
	Mock (control)	CRM197(100 μg/mL)
RK3E	0.524 ± 0.078	0.409 ± 0.055
V5‐1‐NAC‐1 RK3E cells	31.337 ± 0.535	6.587 ± 0.122* (4.76)
V5‐2‐NAC‐1 RK3E cells	30.907 ± 0.489	6.715 ± 0.145* (4.60)
V5‐3‐NAC‐1 RK3E cells	30.343 ± 0.417	6.539 ± 0.138* (4.64)

The fold reversal of drug resistance (number in parentheses) = IC_50(treatments)_/IC_50(control)_. Data are presented as mean ± SD from three independent experiments.

* *P* < .01 vs the V5‐NAC‐1 RK3E cells with mock treatment.

### 3.5 NAC‐1 expression regulates the CRM197‐mediated p38MAPK/JNK pathway via Gadd45gip1/Gadd45γ

**Figure 5:**
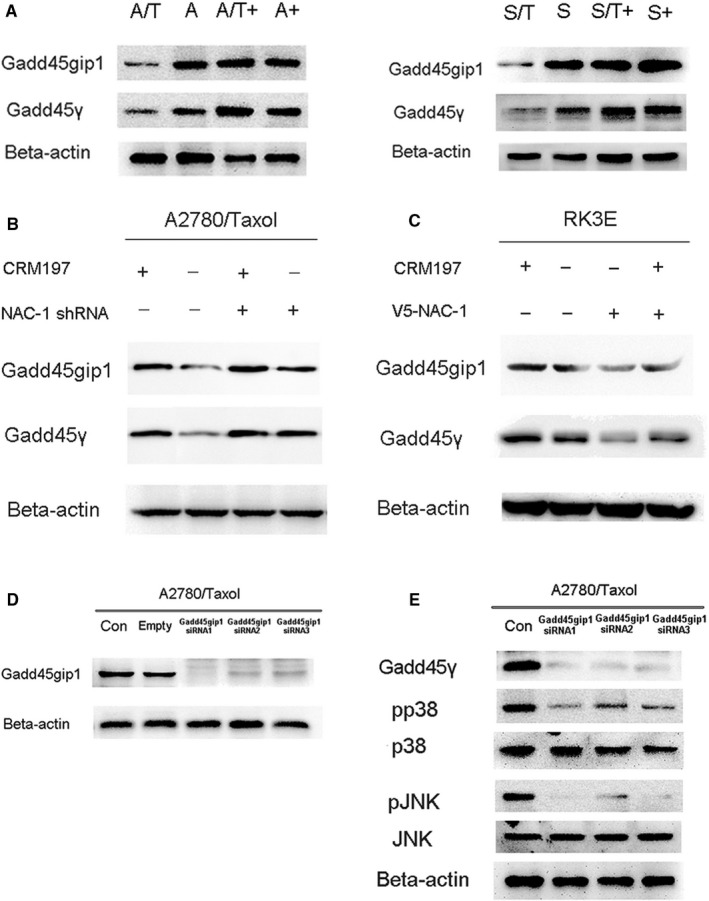
CRM197 decreases NAC‐1 expression by upregulation of the Gadd45gip1/Gadd45γ pathway in paclitaxel‐resistant ovarian cancer cells. A, CRM197 increased expression of Gadd45gip1 and Gadd45γ in parental (A2780 and SKOV3) and paclitaxel‐resistant (A2780/Taxol and SKOV3/Taxol) ovarian cancer cells. B, Expression levels of Gadd45gip1 and Gadd45γ were detected in A2780/Taxol cells treated with NAC‐1 shRNA (NAC‐1 shRNA1) or control shRNA by western blotting (*P* < .01). C, Expression levels of Gadd45gip1 and Gadd45γ were detected in RK3E cells transfected with the V5‐NAC‐1 vector (V5‐1) or control vector by western blotting (*P* < .01). D, A2780/Taxol cells were transiently transfected with Gadd45gip1 siRNA (Gadd45gip1 siRNA1, siRNA2, and siRNA3) or the scramble empty vector (Empty), or treated with PBS (Con, control). Western blotting for Gadd45gip1 was performed to detect the inhibition efficiency of siRNA. E, Expression levels of Gadd45γ and activation of p38MAPK/JNK were detected in A2780/Taxol cells transfected with Gadd45gip1 siRNA (Gadd45gip1 siRNA1, siRNA2, and siRNA3) or control siRNA

The Gadd45gip1/Gadd45γ pathway is involved in tumor recurrence as a downstream pathway of NAC‐1.^17^, ^18^ To investigate whether CRM197 reverses resistance to paclitaxel via the Gadd45gip1/Gadd45γ pathway, we examined the effect of CRM197 on the expression of Gadd45gip1/Gadd45γ in parental (A2780 and SKOV3) and paclitaxel‐resistant (A2780/Taxol and SKOV3/Taxol) ovarian cancer cells by western blotting. As shown in Figure 5A, CRM197 significantly upregulated the expression of Gadd45gip1 and Gadd45γ in A2780/Taxol and SKOV3/Taxol cells (*P* < .01), suggesting that expression of Gadd45gip1/Gadd45γ was involved in the effects of CRM197. To elucidate the effect of the NAC‐1 downstream pathway Gadd45gip1/Gadd45γ on CRM197‐mediated reversal of paclitaxel resistance, we detected the expression of Gadd45gip1/Gadd45γ in NAC‐1 shRNA A2780/Taxol cells (Figure 5B) and V5‐NAC‐1 RK3E cells (Figure 5C). Expression of Gadd45gip1/Gadd45γ in NAC‐1 shRNA A2780/Taxol cells was significantly higher than that in control shRNA A2780/Taxol cells, whereas the expression of Gadd45gip1/Gadd45γ in V5‐NAC‐1 RK3E cells was significantly lower than that in control RK3E cells (both *P* < .01, Figure 5B,C).

**Figure 6:**
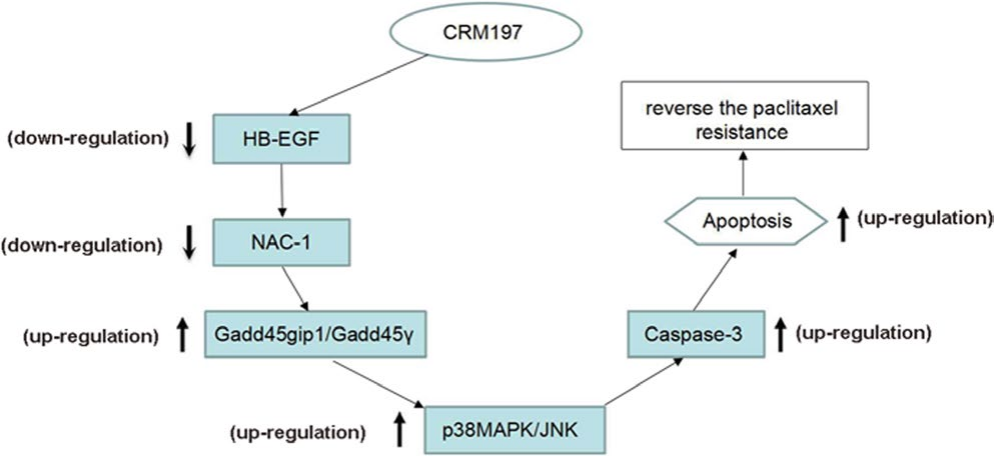
Scheme illustrating the possible mechanisms of CRM197‐mediated reversal of resistance against paclitaxel by inhibiting NAC‐1 in paclitaxel‐resistant ovarian cancer cells

Further investigation of the relationships among Gadd45gip1, Gadd45γ, and P38/JNK was performed in A2780/Taxol cells. We knocked down Gadd45gip1 in A2780/Taxol cells using Gadd45gip1 shRNA (Gadd45gip1 shRNA1, shRNA2, and shRNA3; Figure 5D, *P* < .01) and observed a decrease in the Gadd45gip1 expression level with concomitant decreases in the levels of both Gadd45γ and p38MAPK/JNK (Figure 5E, *P* < .01), suggesting that expression of Gadd45γ and p38MAPK/JNK is dependent on Gadd45gip1 expression. Taken together, these data demonstrated that CRM197 markedly downregulates the NAC‐1 expression by upregulating the Gadd45gip1/Gadd45γ/p38MAPK/JNK pathway in A2780/Taxol cells (Figure 6).

## 4 DISCUSSION

CRM197 is currently the only known HB‐EGF inhibitor that can be used for cancer therapies in mice and humans. We and others have shown that CRM197 is a promising chemotherapeutic and chemosensitizing agent with potent in vitro and in vivo antitumor activities and the ability to reverse resistance against paclitaxel in ovarian cancer.^4^, ^10^ Yagi et al showed that the combination of paclitaxel with CRM197 inhibited cell proliferation and enhanced apoptosis by both inhibiting the activation of ERK and Akt, and stimulating the activation of p38 and JNK.^19^ However, the exact mechanisms by which CRM197 reverses resistance to paclitaxel were unclear, and the interactions associated with regulation between CRM197 and NAC‐1 in paclitaxel resistance of ovarian carcinoma were unknown. In this study, we analyzed the mechanism of reversion of paclitaxel resistance by CRM197 and identified a signaling axis involving NAC‐1/Gadd45gip1/Gadd45γ, which appears to enhance activities of the proapoptotic JNK/p38MAPK pathway that induces paclitaxel resistance in ovarian cancer.

NAC‐1, a transcriptional repressor that belongs to the BTB/POZ gene family, has an oncogenic potential and significant overexpression in several types of human carcinomas including ovarian cancer.^18^, ^20^ Intense NAC‐1 immunoreactivity in primary ovarian tumors predicts early recurrence after chemotherapy, suggesting that NAC‐1 is a tumor recurrence‐associated gene and contributes to the pathogenesis of cancers.^18^ Nevertheless, except for its role in cocaine‐induced expression in animal brains, the mechanisms by which NAC‐1 promotes survival of tumor cell and confers resistance to chemotherapy remain largely unclear.

Here, we found that NAC‐1 contributes to chemoresistance against paclitaxel in epithelial ovarian cancer cells. Similarly, emerging evidence implies that NAC‐1 antagonizes the antitumor activity of conventional chemotherapeutic agents, suggesting that NAC‐1 is a chemoresistance‐associated gene. By correlating NAC‐1 expression and CRM197‐mediated reversal of paclitaxel resistance, we further found that CRM197 reverses resistance to paclitaxel by inhibiting NAC‐1 expression, suggesting a possible mechanism for the reversal of chemotherapy resistance mediated by CRM197.

GADD45 is a tumor suppressor and essential player in the initiation and progression of malignancies.^20^ Accumulated evidence suggests that induction of GADD45 expression is an essential step to mediate anticancer activity of multiple chemotherapeutic drugs, and the absence of GADD45 might abrogate their effects in cancer cells.^20^, ^21^ NAC‐1 controls cell growth and survival by inactivating the Gadd45gip1/Gadd45 pathway in ovarian cancer.^22^ The major property of Gadd45 family proteins is that overexpression of each individual Gadd45 protein induces apoptosis‐related activity and causes apoptosis.^23^, ^24^ In this study, the Gadd45gip1/Gadd45γ pathway as the NAC‐1 downstream signaling pathway contributed to reversal of paclitaxel resistance by CRM197 via modulating the proapoptotic JNK/p38MAPK pathway, which may represent possible mechanisms for the reversal of paclitaxel resistance by CRM197.

Cancer mortality and morbidity are related to recurrence and drug resistance. A positive correlation between NAC‐1, a tumor recurrence‐associated gene, and CRM197, which is a promising chemotherapeutic and chemosensitizing agent, in ovarian carcinoma as shown in this study has significant biological and clinical implications. However, the lack of a correlation between NAC‐1 expression and in vitro drug resistance against paclitaxel suggested that NAC‐1 may not be the only target in the effect of CRM197 on drug resistance. Further study on how CRM197 reverses paclitaxel resistance by NAC‐1 is currently underway in our laboratory.

In this study, NAC‐1 was highly expressed in paclitaxel‐resistant ovarian cancer cells and tissues, which was linked to clinical tumor recurrence and implicated in the reversion of paclitaxel resistance by CRM197. We also found that NAC‐1 activates the proapoptotic JNK/p38MAPK pathway, resulting in apoptosis and induction of resistance to paclitaxel by modulating its downstream pathway, Gadd45gip1/Gadd45, in ovarian cancer cells. Most importantly, this is the first study demonstrating the contribution of NAC‐1 to reversion of paclitaxel resistance by CRM197 in ovarian cancer cells. Our previous and present studies suggest that CRM197 may improve the treatment efficacy of conventional paclitaxel‐based chemotherapy in patients with paclitaxel‐resistant ovarian cancer. This study provides the first mechanism through which CRM197 significantly reverses resistance to paclitaxel by modulating the NAC‐1/Gadd45gip1/Gadd45 pathway in paclitaxel‐resistant ovarian cancer cells and tissues, and HB‐EGF as a possible novel therapeutic target for patients with paclitaxel‐resistant ovarian cancer.

## CONFLICT OF INTEREST

The authors declare that they have no competing interests.

## Data Availability

This is an open access article under the terms of the Creative Commons Attribution License, which permits use, distribution and reproduction in any medium, provided the original work is properly cited.
